# Xylanase supplementation in energy-deficient corn-based diets: impact on broiler growth, nutrient digestibility, chyme viscosity and carcass proximates

**DOI:** 10.5713/ab.23.0340

**Published:** 2024-04-01

**Authors:** Bernadette Gerpacio Sta. Cruz, Jun Seon Hong, Myunghwan Yu, Elijah Ogola Oketch, Hyeonho Yun, Dinesh D. Jayasena, Jung-Min Heo

**Affiliations:** 1 Department of Animal Science and Biotechnology, Chungnam National University, Daejeon 34134, Korea; 2 Technical Marketing, Protein Solution Division, CJ CheilJedang Bio, Seoul 04560, Korea; 3 Department of Animal Science, Uva Wellassa University, Badulla 90000, Sri Lanka

**Keywords:** Broilers, Carcass, Digestibility, Energy, Enzyme, Performance

## Abstract

**Objective:**

The goal of the current study was to investigate the impact of various concentrations of xylanase in energy-deficient corn-based diets on the growth performance, carcass characteristics, nutrient digestibility, and digesta viscosity in broilers from 7 to 35 days of age.

**Methods:**

A total of 280 seven-day-old Ross 308 broilers were randomly allocated to one of the five dietary treatments following a completely randomized design with 8 replicates and 7 birds per cage. The treatments were: i) positive control (PC, without xylanase); ii) NC-1 (80 kcal/kg ME reduced from PC); iii) NC-2 (100 kcal/kg ME reduced from PC); iv) NCX-1 (NC-1 + 2,000 U/kg xylanase); and v) NCX-2 (NC-2 + 3,000 U/kg xylanase). Body weight, weight gain, feed intake, and feed conversion ratio were determined weekly to evaluate growth performance. One bird per pen was sacrificed for ileal digesta collection to determine the viscosity and digestibility of energy, dry matter, crude protein on days 24 and 35, however breast and leg meat samples were obtained for proximate analysis (moisture, crude protein, fat, and ash) on day 35.

**Results:**

Birds fed diets supplemented with xylanase regardless of the amount had higher (p<0.05) body weights, daily gains, and improved feed efficiency compared to NC diets all throughout the experimental period. Feed intake was not affected (p>0.05) by the addition of xylanase. Moreover, lowered (p<0.05) viscosity of the ileal digesta were observed upon xylanase inclusion in the diets compared to the birds fed NC diets on day 24. Ileal nutrient digestibility and meat proximate composition were not affected (p>0.05) by xylanase.

**Conclusion:**

The present study indicated that the xylanase at 2,000 U/kg and 3,000 U/kg levels compensates for the 80 kcal/kg and 100 kcal/kg dietary energy levels, respectively, without having adverse effects on the growth performance, carcass characteristics, nutrient digestibility, and digesta viscosity of broilers.

## INTRODUCTION

Maize has been the major energy material used in poultry diets. Recently, shortages in supply and price volatility of corn have become a challenging issue when reducing costs in feed production [1]. Therefore, alternative strategic approaches aimed at the precise utilization of dietary nutrients while considering both the economic and environmental aspects of production have been investigated by the poultry sector. One of these practices includes the supplementation of dietary enzymes for increasing nutrient availability in broiler diets and reducing the detrimental impact of excreta deposition on the environment [2].

Non-starch polysaccharides (NSPs) are anti-nutritional factors commonly found in cereal grains, starch, and other plant-derived ingredients. The cell walls of cereal grains which are commonly used as energy feedstuffs are composed of up to 15% soluble and insoluble NSPs [1]. The insufficient ability of poultry in producing endogenous enzymes for hydrolyzing xylans hinders digestion processes by increasing viscosity and entrapping other nutrients in the intestinal tract, thus affecting nutrient digestibility and growth performance [3]. NSP-hydrolyzing enzymes including xylanases have become commercially available and contribute to more than 50% of the global feed enzyme market. Xylanase splits the 1,4-β-D-xylosidic linkages of the xylan backbone between unsubstituted xylose residues [4]. The dissociation of cell wall components releases energy and amino acids for nutrient utilization in poultry [5]. In addition, oligosaccharides produced during the conversion from arabinoxylans could also exhibit a prebiotic effect which has been reported to improve the growth performance in broiler production [6].

The majority of broiler diets consist of corn-soybean formulation, with corn contributing to about 48.65% of the total arabinoxylan content among NSPs and having relatively higher levels of insoluble than soluble arabinoxylans [7,8]. Although the addition of xylanase to corn-based diets has been applied globally, the response of corn to xylanase is usually less pronounced when compared to wheat, rye, and barley, primarily due to its lower content of NSP [9]. Wheat bran, being a by-product, holds potential as a poultry feed due to its variety of nutrients, such as vitamins and antioxidant compounds [10]. However, the inclusion of wheat bran, which contains approximately 43% to 60% of the total NSP including arabinoxylans, is limited in broiler diets [11,12]. The response of xylanase in diets incorporated with wheat bran has been effective in improving the growth performance and nutrient digestibility in broilers as previously reported [11]. There is still a lack of extensive research on the impact of supplemental xylanase in energy-deficient using corn as the main source of energy in broiler diets. To ascertain the economic viability of using xylanase in broiler diets, further investigations are imperative.

Therefore, the objective of this study was to determine the efficacy of xylanase at 2,000 U/kg and 3,000 U/kg levels in 80 kcal/kg and 100 kcal/kg energy-deficient corn-based diets, respectively. This research tested the hypothesis that xylanase has the potential to mitigate the detrimental impact of energy reduction by lowering intestinal viscosity and improving nutrient digestibility without compromising the performance of broilers. Assessments were conducted for the growth performance, ileal nutrient digestibility, viscosity, and meat-proximate composition of broilers.

## MATERIALS AND METHODS

All experimental procedures of the present study were reviewed and approved according to the guidelines of the Animal Ethics Committee (Protocol No. 202112A-CNU-210), Chungnam National University, South Korea. The xylanase enzyme used in this study was provided by CJ CheilJedang BIO in Seoul, South Korea.

### Birds and housing

A total of 280 7-day-old Ross 308 broiler chicks were randomly allocated in raised wire floor cages (85×55×35 cm^3^) with three nipple drinkers and a metal trough for free access to fresh drinking water and feed. Seven birds were housed per cage. Birds were provided commercial feeds during the pre-experimental period from day 1 to 7; then the experimental diets from day 8 to 35 on an *ad libitum* basis. An ambient temperature of 32°C was lowered by 2 degrees weekly and continuous lighting was provided following the Korean feeding standard for poultry [13]. All other management practices were also implemented using these guidelines.

### Experimental design and management

Birds were randomly assigned to one of the five dietary treatments with eight replicates per treatment using a completely randomized design. Corn and soybean meal diets were formulated based on the Ross 308 Nutritional Specifications [14] listed in Table 1 and 2. The feeding program consisted of a late starter (day 7 to 24) and grower (day 25 to 35) phase and was provided both in mash form. The dietary treatments were as follows: i) positive control (PC, without xylanase); ii) NC-1 (80 kcal/kg ME reduced from PC); iii) NC-2 (100 kcal/kg ME reduced from PC); iv) NCX-1 (NC-1 + 2,000 U/kg xylanase); and v) NCX-2 (NC-2 + 3,000 U/kg xylanase). Xylanase was added to the broiler feeds through a premix, which was subsequently blended with the remaining feeds. Moreover, Cr_2_O_3_ (Chromium oxide powder, >99.9% purity; Sigma Aldrich, St. Louis, MO, USA) was added to the diets to serve as an internal indigestible marker in analyzing the digestibility of nutrients.

### Growth performance evaluation

During the entire experimental period, the average body weight (BW) and feed remains of the birds were recorded weekly on a pen basis to determine the average daily gain (ADG) and average daily feed intake (ADFI). Using these data, the feed conversion ratio (FCR) of the birds was also calculated for the entire experimental period. Mortality-adjusted FCR was also determined in cases of mortalities.

### Post-mortem procedure and sample collection

Sample collections of digesta were acquired on days 24 and 35 for the ileal nutrient digestibility and viscosity analysis. One bird closest to the mean BW per cage was selected and euthanized by cervical dislocation. Abdominal incisions on the bird were made to remove the ileum and jejunum from the gastrointestinal tract for collecting digesta. Ileum and jejunum were differentiated through the location of Meckel’s diverticulum. The segment of the small intestine that extends from Meckel’s diverticulum to the ileocecal junction is the ileum. The ileum was separated from the jejunum by cutting 3 cm from the Meckel’s diverticulum. Thereafter, digesta from the ileal was collected through gentle flushing with distilled water into a labeled plastic container as conducted by Oketch et al [15]. Samples were then freeze-stored at −20°C until further analysis.

Breast and leg muscle samples were collected for proximate composition analysis on day 35. Breast and drumsticks from the leg muscle were extracted, deboned, and placed in resealable plastic bags. The samples were then sent to the laboratory for further analysis.

### Sample preparation and laboratory analysis

Digesta viscosity was determined based on the method of Liu and Kim [16]. Collected samples of the ileal digesta from each pen were centrifuged a 4,000 rpm at 4°C for 15 mins. Each tube containing 0.5 mL supernatant was analyzed for viscosity using a viscometer (Model DV-II; Brookfield, Middleboro, MA, USA). The unit used to express viscosity measurement is mPa/s.

The rate of nutrient disappearance at the terminal ileum was estimated by determining the apparent ileal digestibility (AID) of energy, dry matter, and crude protein (CP). Collected digesta samples were dried at 55°C for 24 h, grounded, and strained through a 0.75 mm sieve (ZM 200 Ultra-Centrifugal Mill; Retsch GmbH & Co. KG, Haan, Germany). Nutrient digestibility was determined through the calculation of the chromium oxide concentration in the digesta using the method of Fenton and Fenton [17]. AID was computed using the equation below where M_diet_ refers to the concentration of the indigestible marker in the diet, N_digest_ as the nutrient concentration in ileal digesta, M_digest_ as the concentration of the indigestible marker in ileal digesta, and N_diet_ as the nutrient concentration in the diet [15].


$$\%\;\text{Digestibility}=100-[100\times \left(\frac{M_{Diet}\times N_{Digest}}{M_{Digest}\times N_{Diet}}\right)]$$

Concerning the carcass characteristics, the moisture, CP, crude fat, and ash of the breast and leg meat samples were determined according to the method used by Latimer [18] and Premathilaka et al [19]. Moisture content was obtained by removing the water by drying it in an oven. Two grams of meat per sample was placed in Petri dishes, and then dried in an oven at 103°C for 16 to 18 h. After drying, the Petri dishes with the samples were cooled and recorded for weight. The moisture content was then calculated using the equation below where W_1_ is the weight of the petri dish, W_2_ is the weight of the sample in a petri dish before drying and W_3_ is the weight of the sample in a petri dish after drying.


$$\%\;\text{Moisture}=\left(\frac{W_{3}-W_{1}}{W_{2}-W_{1}}\right)\times 100$$

Crude protein was determined using the Kjeldahl procedure according to the AOAC method [18]. Percentage nitrogen was obtained through titration first and was later used to calculate the CP using the formula as follows:


% Crude protein=% N×6.25

Crude fat was determined by using the Soxhlet extraction method according to AOAC [18]. Five grams of meat samples were extracted for 8 h through the soxhlet apparatus (DKZW-4; Shanghai Kexi Instrument LTD, Shanghai, China) and were then calculated using the equation below where W_1_ is the weight of the dried sample, W_2_ is the weight of the water’s volume in the flask and W_3_ is the volume of water after fat extraction in the flask.


$$\%\;\text{Fat}=\left(\frac{W_{3}-W_{2}}{W_{1}}\right)\times 100$$

In obtaining the ash content, 3 g of the meat samples were placed first into crucibles. The crucibles were dried and then ignited in a muffle furnace (HD 230 PAD; Horno de Mufla, Tecny lab, Burgos, Spain) at 550°C for 4 h until a light grey ash was obtained. After turning it into an ash-dried sample, the weight was recorded. Ash content was computed using the equation below where W_1_ was the weight of the crucible without the sample, W_2_ was the weight of the crucible with the dried sample before ignition, and W_3_ was the weight of the crucible containing the ash after ignition.


$$\%\;\text{Ash}=\left(\frac{W_{3}-W_{1}}{W_{2}-W_{1}}\right)\times 100$$

### Statistical analyses

Data were analyzed using a general linear model procedure for one-way analysis of variance test in a completely randomized design through the SPSS 26.0 (SPSS Inc., Chicago, IL, USA) program. A pen was used as the experimental unit for the parameters on growth performance (BW, ADG, ADFI, and FCR) whereas selected individual birds were used for sample collection as an experimental unit for meat nutrient composition, digesta viscosity, and ileal nutrient digestibility measurements. Tukey’s multiple range test was performed to identify significant variations between treatments at a 95% confidence level.

## RESULTS

### Growth performance

Body weight of the birds fed dietary treatments PC and NCX-2 were consistently higher (p<0.05) than those fed the other diets throughout the experimental period (d 14, 21, 24, 28, and 35; Table 3). Concerning ADG, birds fed xylanase and the PC gained higher daily weights (p<0.05) than those fed NC diets on the starter period (day 7 to 24), and over the entire experimental period (day 7 to 35).

Average daily feed intake was neither affected significantly nor marginally (p>0.05) upon xylanase supplementation during the entire experimental period. Concerning feed efficiency, birds fed PC diets improved (p<0.05) in the recorded FCR values than all the negative control diets throughout the experimental period. In comparison to the negative control diets, NCX-1 and NCX-2 had lower values that significantly improved feed efficiency (p<0.05) than the other negative control diets during the entire experimental period (day 7 to 35).

### Ileal viscosity and nutrient digestibility

Viscosity and ileal nutrient digestibility measurements of broilers are recorded in Table 4. Xylanase-supplemented diets regardless of dosage reduced viscosity (p<0.05) in the intestinal tract more than in the birds in NC on day 24. Concerning nutrient digestibility, no significant effects (p>0.05) were noted for the values recorded for the energy, dry matter, and protein on days 24 and 35.

### Meat proximate composition

The influence of meat proximate composition upon xylanase supplementation is recorded in Table 5. Birds assigned to diets with xylanase did not show any differences (p>0.05) in the breast and leg meat composition in terms of moisture, CP, fat, and ash content between treatments.

## DISCUSSION

Corn contains relatively lower NSP concentrations, particularly arabinoxylans, in comparison to wheat and other cereal grains [20]. The performance risks associated with feeding broilers high NSP grain by-products in energy-reduced corn-based diets were mitigated through xylanase supplementation, as previously reported [2]. In the present study, wheat bran was incorporated into the diets to elevate the total NSP content in the diets. According to Jaworski et al [20], arabinoxylans makes up to 64.3% of the total NSP in wheat bran. Wheat bran in the current study was included in the diets to enhance the response of xylanase while mitigating the energy deficit in broiler diets. Therefore, the present study was designed to supplement xylanase at 2,000 U/kg and 3,000 U/kg levels in 80 kcal/kg and 100 kcal/kg reduced-energy diets, respectively. As expected, broilers fed energy-deficient diets without xylanase exhibited lower growth performance compared to diets formulated with adequate energy, aligning with findings reported by Saleh et al [21] and Ismael et al [22], where reduced-energy diets of 90 kcal/kg and 120 kcal/kg negatively impacted broiler growth performance, respectively. The inability of broilers to produce NSP hydrolyzing enzymes allows xylans to pass through the colon almost undigested, thereby increasing digesta viscosity that encapsulates nutrients, modulates gut microflora, and shortens digesta transit time. Consequently, this depresses absorption and reduces nutrient digestibility, potentially negatively affecting growth performance [3,7].

Upon supplementation of xylanase in the negative control diets, the present study demonstrated that xylanase at 2,000 U/kg and 3,000 U/kg levels were able to compensate for the reduced energy in the diets by showing comparable results with the PC diet, increased BW, and ADG, and improved FCR than those fed a diet without xylanase. The principle behind the improvements in growth performance is probably due to the hydrolyzing action of xylanase on the 1,4-β-D-xylosidic linkages of arabinoxylans (AX) [7]. These backbones are randomly cleaved by xylanase, resulting in the production of xylooligosaccharides including xylose and other simple sugars; that are later converted as energy nutrients making it readily available for broilers; thus, making up for the reduced energy in the diet [2,23,24]. Furthermore, xylanase has been reported to lack the ability to influence the feed intake of broilers as was observed in the present study and reported in previous literature [23,25].

Xylanase could also be more effective in improving the microbiota profile of younger broilers as they neither have a completely developed gastrointestinal tract nor an established microbiota [2]. The present study agrees with this observation as higher ADG was shown in birds assigned with xylanase than those with negative control diets during the earlier stage. Nusairat and Wang [23] also reported that broilers fed with 15,000 XU/kg xylanase level in 130 kcal/kg energy-deficient diets showed higher BW gain than negative control diets from hatch to 21 d post-hatch. Degradation of xylans allows the production of xylo-oligomers which establishes more beneficial bacteria responsible for gut fermentation in the ceca [6]. Although the microbiota profile was not evaluated in the present study, Nian et al [26] demonstrated that the addition of xylanase in corn-based diets increased the population count of *lactobacillus* and *bifidobacterial* in the cecal contents. An incremental increase in the beneficial bacteria population leads to improved performance due to their mode of action on the competitive exclusion of pathogenic bacteria, synthesis of antimicrobial substances, and improvements in the immune system and intestinal morphology [27].

Birds lack endogenous enzymes to hydrolyze NSPs including xylans that are responsible for increased digesta viscosity and reduced nutrient digestibility; due to the caging effect of NSPs on nutrients in the small intestine [7]. A couple of studies have highlighted that the addition of xylanase to cereal grains, especially wheat, has reduced the intestinal digesta viscosity in broilers [16,28]. However, Khadem et al [29] demonstrated that xylanase has no influence on the digesta viscosity of broilers fed corn-soybean meal diets. The xylanase effect should be more evident in wheat than corn, given that wheat has higher arabinoxylans content (7.3% vs 4.7%). This leads to greater availability of substrates for increasing xylanase activity [30,31]. The study findings indicated a reduction in the viscosity of broiler intestinal digesta when xylanase was introduced to the diet containing wheat bran, resembling the findings of Kiarie et al [9], where corn dry distillers grains with solubles with 12% insoluble arabinoxylans led to a decrease in broiler intestinal viscosity. The reduction of digesta viscosity is probably due to the hydrolytic action of xylanase on the highly branched arabinoxylans, thereby releasing trapped nutrients [32]. Moreover, the present results showed that the effect of xylanase on the ileal viscosity was more evident during the earlier phase (i.e., late starter). The effects of exogenous enzymes could be associated with age, more so in the earlier growth stages because young broilers lack sufficient enzymes to hydrolyze fibers and non-carbohydrate polymers in cereal grains [23,32].

In the present study, lower digesta viscosity was observed upon the inclusion of xylanase in diets. However, the observation did not show any effect on the ileal nutrient digestibility for energy, dry matter, or protein, similar to the findings of Kiarie et al [9]. Exogenous enzyme action in degrading NSPs occurs at the ileal and cecal stages. Enzymes eliminate fermentable substrates during the ileal phase whereas xylose and xylo-oligomers generated from the NSP degradation are fermented by bacteria in the cecal phase. This promotes the growth of beneficial bacteria and the production of volatile fatty acid (VFA) including butyric acid [33,34]. Volatile fatty acids generated in the ceca could contribute approximately 3% to 5% of the total energy requirements for broilers [9]. Furthermore, it was observed that VFAs can stimulate a neuro-hormonal reaction that triggers an ‘ileal break,’ meaning it delays gastric emptying and gut motility, thus, extending the metabolic site for digestion from the proximal to a more distal region of the gastrointestinal tract [9,34]. The rate of nutrient digestibility could be perceived on an ileal or even total tract basis that includes liver wastes and distal gut microflora effects [35]. Therefore, nutrients generated through VFA production and fermentation may have also contributed to the enhancement of growth performance in broilers fed with xylanase in the present study, despite not having any effect on digestibility in the ileal intestinal tract. This observation is consistent with the findings reported by Williams et al [36] where nutrient digestibility was not affected but showed superior growth performance upon xylanase supplementation in broilers. Associating the ileal nutrient digestibility with growth performance and digesta viscosity in broilers could be misleading since it could be affected by the age of birds, raw materials used, and different sites for nutrient digestion.

The present study did not show any improvements in the breast and leg meat on moisture, CP, fat, and ash composition upon xylanase supplementation individually or in combination with other enzymes as has been previously reported [37]. It is plausible that improvements in carcass nutrient composition and parameters of meat proximate composition could be achieved if other feed additives are combined with xylanase in the broiler diets [38]. To date, there is limited literature on the effects of xylanase on the meat proximate composition and muscle quality of broilers based solely on xylanase. Further studies that elucidate the potential effects of xylanase on meat quality are crucial.

## CONCLUSION

Supplementation of xylanase at 2,000 U/kg and 3,000 U/kg levels could be effective in the decomposition of NSP to compensate for the reduced 80 kcal/kg and 100 kcal/kg dietary energy in broiler diets without compromising broiler performance, respectively. The influence of xylanase on ileal nutrient digestibility was not observed in the study; however, improvements in growth performance might be attributed to the reduced digesta viscosity, indicating a potential increase in favorable gut microorganisms and enhanced nutrient digestion due to the production of VFA in the cecum. Meat proximate composition was not affected by xylanase supplementation.

## Figures and Tables

**Table 1:** Composition (as-fed basis) of experimental starter diets

Items	Diets		

	PC	NC-1	NC-2
Ingredients (%)			
Corn	57.46	55.71	55.28
Wheat bran	4.00	6.67	7.34
SBM, 48 %	30.25	29.54	29.36
Fish meal	1.90	2.03	2.07
Vegetable oil	2.35	1.85	1.73
Limestone	1.08	1.27	1.32
Mono-calcium P	1.52	1.51	1.51
Salt	0.30	0.30	0.30
Vit-Min premix^1)^	0.30	0.30	0.30
Lys-HCI	0.25	0.25	0.25
DL-methionine	0.29	0.28	0.28
Cr_2_O_3_	0.30	0.30	0.30
Calculated composition			
Metabolized energy (kcal/kg)	3,050	2,970	2,950
Crude protein (%)	21.50	21.50	21.50
Non-starch polysaccharides (%)	9.07	9.66	9.80
Lysine (%)	1.32	1.32	1.32
Methionine+cysteine (%)	1.00	0.99	0.99
Methionine (%)	0.64	0.64	0.64
Calcium (%)	0.96	0.96	0.96
Available phosphorus (%)	0.50	0.51	0.51

1) Vitamin and mineral mixture provided the following nutrients per kg of diet: vitamin A, 24,000 IU; vitamin D_3_, 6,000 IU; vitamin E, 30 IU; vitamin K, 4 mg; thiamin, 4 mg; riboflavin, 12 mg; pyridoxine, 4 mg; folacine, 2 mg; biotin, 0.03 mg; vitamin B_8_ 0.06 mg; niacin, 90 mg; pantothenic acid, 30 mg; Fe, 80 mg (as FeSO_4_·H_2_O); Zn, 80 mg (as ZnSO_4_·H_2_O); Mn, 80 mg (as MnSO_4_·H_2_O); Co, 0.5 mg (as CoSO_4_·H_2_O); Cu, 10 mg (as CuSO_4_·H_2_O); Se, 0.2 mg (as Na_2_SeO_3_); I, 0.9 mg (as Ca (IO_3_)·2H_2_O).

**Table 2:** Composition (as-fed basis) of experimental grower diets

Items	Diets		

	PC	NC-1	NC-2
Ingredients (%)			
Corn	51.35	54.02	54.69
Wheat bran	4.00	4.67	4.84
SBM, 48 %	27.87	27.34	27.21
Fish meal	2.20	2.07	2.04
Vegetable oil	3.30	1.94	1.60
Corn starch	7.00	5.77	5.47
Limestone	1.38	1.28	1.26
Mono-calcium P	1.50	1.50	1.50
Salt	0.30	0.30	0.30
Vit-Min premix^1)^	0.30	0.30	0.30
Lys-HCI	0.20	0.22	0.23
DL-methionine	0.30	0.30	0.30
Cr_2_O_3_	0.30	0.30	0.30
Calculated composition			
Metabolized energy (kcal/kg)	3,150	3,070	3,050
Crude protein (%)	20.00	20.00	20
Non-starch polysaccharides (%)	8.35	8.65	8.72
Lysine (%)	1.22	1.22	1.22
Methionine+cysteine (%)	0.96	0.97	0.97
Methionine (%)	0.63	0.63	0.63
Calcium (%)	1.04	1.04	1.03
Available phosphorus (%)	0.49	0.49	0.49

1) Vitamin and mineral mixture provided the following nutrients per kg of diet: vitamin A, 24,000 IU; vitamin D_3_, 6,000 IU; vitamin E, 30 IU; vitamin K, 4 mg; thiamin, 4 mg; riboflavin, 12 mg; pyridoxine, 4 mg; folacin, 2 mg; biotin, 0.03 mg; vitamin B_8_ 0.06 mg; niacin, 90 mg; pantothenic acid, 30 mg; Fe, 80 mg (as FeSO_4_·H_2_O); Zn, 80 mg (as ZnSO_4_·H_2_O); Mn, 80 mg (as MnSO_4_·H_2_O); Co, 0.5 mg (as CoSO_4_·H_2_O); Cu, 10 mg (as CuSO_4_·H_2_O); Se, 0.2 mg (as Na_2_SeO_3_); I, 0.9 mg (as Ca (IO_3_)·2H_2_O).

**Table 3:** Effects of xylanase on growth performance in broilers^1)^

Items	Dietary treatments^2)^					SEM^3)^

	PC	NC-1	NC-2	NCX-1	NCX-2	
Body weight (g)						
d 7	136.55	135.54	134.03	135.96	136.22	0.543
d 14	361.66^b^	342.07^ab^	329.75^a^	357.44^b^	364.23^b^	8.134
d 21	701.22^b^	635.78^a^	606.71^a^	665.41^ab^	696.07^b^	24.012
d 24	982.02^c^	902.81^ab^	870.28^a^	949.77^bc^	977.99^c^	24.346
d 28	1,338.63^b^	1,248.20^ab^	1,212.45^a^	1,311.36^b^	1,329.30^b^	33.083
d 35	1,910.78^c^	1,779.88^ab^	1,776.43^a^	1,895.29^abc^	1,908.34^bc^	45.142
Average daily gain (g/d)						
d 7 to 24	49.73^c^	45.13^ab^	43.31^a^	47.87^bc^	49.52^bc^	1.435
d 25 to 35	84.43	79.73	82.38	85.96	84.58	3.103
d 7 to 35	63.37^b^	58.73^a^	58.66^a^	62.83^b^	63.29^b^	1.619
Average daily feed intake (g/d)						
d 7 to 24	68.52	67.22	66.55	67.14	68.68	0.437
d 25 to 35	120.99	127.01	132.65	124.89	125.34	1.798
d 7 to 35	89.01	90.72	92.47	89.80	90.77	0.549
Feed conversion ratio (g/g)						
d 7 to 24	1.38^a^	1.49^b^	1.54^bc^	1.40^a^	1.39^a^	0.031
d 25 to 35	1.43^a^	1.59^b^	1.61^b^	1.45^a^	1.48^a^	0.043
d 7 to 35	1.40^a^	1.54^b^	1.58^b^	1.43^a^	1.43^a^	0.037

1) Values are the mean of eight replicates per treatment.

2) PC, positive control diet; NC-1, 80 kcal/kg energy-deficient level diet; NC-2, 100 kcal/kg energy-deficient level diet; NCX-1, NC-1 with 2,000 U/kg xylanase; NCX-2, NC-2 with 3,000 U/kg xylanase.

3) Pooled standard error of mean.

a–c Values in a row with different superscripts differ significantly (p<0.05).

**Table 4:** Effects of xylanase on ileal viscosity and ileal nutrient digestibility of broilers^1)^

Items	Dietary treatments^2)^					SEM^3)^

	PC	NC-1	NC-2	NCX-1	NCX-2	
Viscosity (mPa/s)						
d 24	2.64^ab^	2.73^a^	2.73^a^	2.56^bc^	2.50^c^	0.039
d 35	2.55^ab^	2.61^a^	2.58^ab^	2.52^ab^	2.46^b^	0.043
Energy digestibility (%)						
d 24	84.62	83.72	81.65	81.64	79.99	2.075
d 35	84.95	84.66	83.97	83.06	83.71	1.091
Dry matter digestibility (%)						
d 24	85.84	84.21	82.22	83.59	80.88	1.661
d 35	86.32	85.30	84.68	85.06	83.61	0.978
Crude protein digestibility (%)						
d 24	83.71	82.61	79.97	80.47	79.46	1.990
d 35	84.03	83.55	82.95	82.19	82.73	1.088

1) Values are the mean of eight replicates per treatment.

2) PC, positive control diet; NC-1, 80 kcal/kg energy-deficient level diet; NC-2, 100 kcal/kg energy-deficient level diet; NCX-1, NC-1 with 2,000 U/kg xylanase; NCX-2, NC-2 with 3,000 U/kg xylanase.

3) Pooled standard error of mean.

a–c Values in a row with different superscripts differ significantly (p<0.05).

**Table 5:** Effects of xylanase on meat proximate composition of broilers^1)^

Nutrient	Dietary treatments^2)^					SEM^3)^

	PC	NC-1	NC-2	NCX-1	NCX-2	
Breast meat (%)						
Moisture	72.67	72.07	74.22	73.11	73.59	0.743
Crude protein	22.96	23.25	22.79	23.91	23.81	0.495
Crude fat	1.70	1.32	1.21	2.13	1.27	0.605
Ash	1.62	1.60	1.54	1.56	1.51	0.121
Leg meat (%)						
Moisture	75.15	75.06	73.94	74.06	74.37	0.958
Crude protein	18.32	19.10	18.24	19.70	19.51	0.530
Crude fat	5.36	3.88	4.02	5.47	4.39	1.189
Ash	1.26	1.32	1.43	1.39	1.47	0.131

1) Values are the mean of eight replicates per treatment.

2) PC, positive control diet; NC-1, 80 kcal/kg energy-deficient level diet; NC-2, 100 kcal/kg energy-deficient level diet; NCX-1, NC-1 with 2,000 U/kg xylanase; NCX-2, NC-2 with 3,000 U/kg xylanase.

3) Pooled standard error of mean.

Statistical significance was determined at p<0.05.
